# 541. Comparing Patients with Severe COVID-19 Who Improve to the Point of Discharge Following an Abbreviated Course (< 5 Days) of Remdesivir (RDV) Versus a Standard Course (≥5 Days)

**DOI:** 10.1093/ofid/ofab466.740

**Published:** 2021-12-04

**Authors:** Huan Pham, Qiaoling Chen, Aldon Li, Adam Baghban, Anita Cheruvanky, Graciela Faiad

**Affiliations:** 1 Kaiser Permanente Riverside, Riverside, California; 2 Kaiser Permanente Southern California, Pasadena, California; 3 Kaiser Permanente, Riverside, CA

## Abstract

**Background:**

The COVID-19 pandemic has negatively affected our healthcare system. Our hospitals have reached maximum capacity on several occasions. Because of the need to make beds available to new patients, some patients with severe COVID-19 who were on low flow O2 supplementation have been discharged home prior to completion of the standard (≥ 5-day) RDV course. To date, data are limited regarding clinical outcomes on these patients. Because of this, we conducted a retrospective study to assess the clinical outcomes of patients who received an abbreviated treatment course of RDV.

**Methods:**

Retrospective (chart review) study

**Subject population:**

All nonpregnant adult patients who were hospitalized at Kaiser Permanente Riverside Medical Center and Kaiser Permanente Moreno Valley Medical Center in 2020 with severe COVID-19 who required low flow O2 supplement during hospitalization who received RDV and discharged from hospital alive. Severe COVID-19 = positive SARS-CoV-2 PCR + evidence of lung involvement on lung imaging (X-ray or CT) + O2 saturation ≤ 94% on room air or requirement of O2 supplement.

**Inclusion criteria:**

Age ≥ 18 years; Hospitalized with severe COVID-19; Given RDV

**Exclusion criteria:**

Pregnancy; O2 requirement > 6 L including high flow and mechanical ventilation (noninvasive or invasive); discontinuation of RDV due to adverse effects

Figure 1. Patient Section.

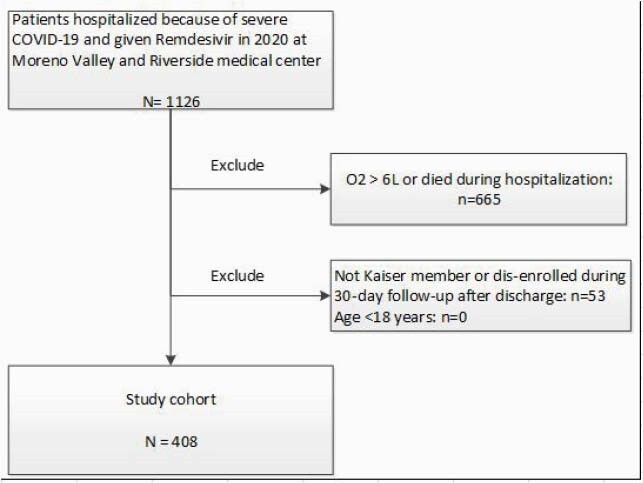

**Results:**

Mortality rate: no difference (2.1% vs 1.8%, p=0.84). 30 day post-discharge ED visit: twice more likely in the abbreviated RDV group as compared to the group receiving the standard duration (16.1% vs 8.5%, p=0.03). 30 day readmission: almost 10 times more likely in the abbreviated RDV group as compared to the group receiving the standard duration (11.9% vs 1.2%, p=< 0.001).

Table 1. Patient's Characteristics

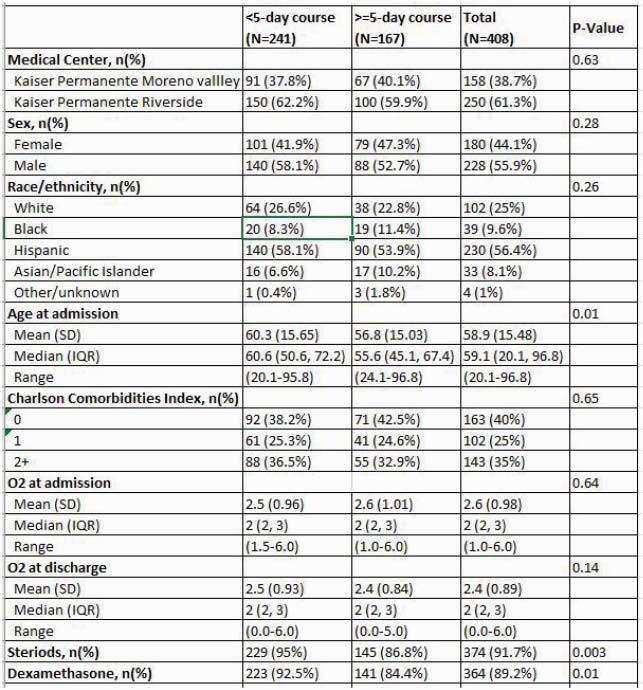

Table 2. Clinical Outcomes. *8 Patients Who Died Within 30-Day from Discharge Were Excluded

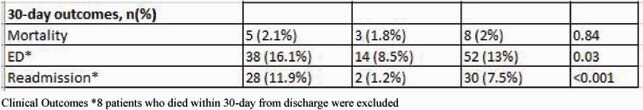

**Conclusion:**

Though there is no difference in 30 day mortality rate, the patients who received the abbreviated RDV course are twice more likely to have ER visit and 10 times more likely to have readmission within 30 day post discharge despite more patients in the abbreviated course receiving steroids. The findings suggest that completing an at least 5-day course of RDV may be beneficial even in patients who demonstrate a clinical response earlier in course.

**Disclosures:**

**All Authors**: No reported disclosures

